# Clustering- and Transformer-Based Networks for the Style Analysis of Logo Images

**DOI:** 10.1155/2022/2090712

**Published:** 2022-05-09

**Authors:** Nannan Tian, Yuan Liu, Ziruo Sun, Xingbo Liu

**Affiliations:** ^1^School of Design, Jiangnan University, Wuxi 214122, China; ^2^School of Artificial Intelligence and Computer Science, Jiangnan University, Wuxi 214122, China; ^3^School of Software Engineering, Shandong University, Jinan 250101, China; ^4^School of Computer Science and Technology, Shandong Jianzhu University, Jinan 250101, China

## Abstract

In the design field, designers need to investigate and collect logo materials before designing logos and search a large number of design materials on well-known logo websites to find logos with similar styles as reference images. However, manual work is time-consuming and labor-intensive. To solve this problem, we propose a clustering method that uses K-Means clustering and visual transformer model to group the styles of the logo database. Specifically, we use the visual transformer model as a feature extractor to convert logo images into feature vectors and perform K-Means clustering, use the clustering results as pseudo-labels to further train the feature extractor, and continue to iterate the above process to finally obtain reliable clustering results. We validate our approach by creating the logo image dataset JN Logo, a proposed database for image quality and style attributes, containing 14922 logo design images. Our proposed deep transformer-based cluster (DTCluster) automatic style grouping method is used in JN Logo; the DBI reaches 0.904, and the DI reaches 0.189, which are better than those of other K-Means clustering methods and other clustering algorithms. We perform a subjective analysis of five features of the clustering results to obtain a semantic description of the clusters. Finally, we provide six styles and five semantic descriptions for the logo database.

## 1. Introduction

Logos are unique, but there are similarities in their style positioning. When designers design a new logo, they need to investigate the logos of similar product industries, Internet industries, service industries, or entity companies as a reference. They manually classify logos based on style, but this method is not universal and is time-consuming. The field of computational aesthetics involves methods of manually labeling logo datasets for classification, but the cost of labor and time is relatively high.

Sign-related studies have been extensively analyzed in fields such as brand infringement detection [1], logo image recognition [2], logo retrieval [3], and logo recognition [4, 5]. As an important branch of logo research, logos are used for detection of video logos [6], logo detection based on convolutional neural network (CNN) [7, 8], and logo dataset [9]. Tian et al. [10] establish a logo dataset for logo image generation in a conditional adversity-generation network. Sage et al. [11], Mino and Spanakis [12] and Oeldorf and Spanakis [13] generate logos mainly based on a generative adversarial network (GAN). There is no standard evaluation system for the performance evaluation of logo image styles in the computing field, but there are relevant books as standards in the design major. These standards are the basic theory and design benchmark for design majors' learning design. They mainly include design chronology, basic theory of logo design, graphic design art books, vision and perception books, logo graphics, and related literature. In this paper, we not only use these evaluation criteria for the algorithm, but also explore the connection between the two based on the algorithm and design theory analysis.

In terms of computational aesthetics, the fields that seek to solve aesthetics include painting, sculpture, ceramics, photography, videography, video, film production, design, handicrafts, and architecture. Fishwick [14] defines “aesthetics” as the combination of cognitive patterns and perceptual patterns. The work in computational aesthetics is to label logo datasets manually to obtain classification methods, but the cost of manpower and time is great.

To solve this challenge, we explored the application of clustering algorithms to find the visual design style of the logo automatically. In recent years, machine learning has become a very good auxiliary tool for artistic creation. Clustering is a designed algorithm that does not rely on objective standards and can find a certain standard that has not been covered by previous algorithms from the dataset angle.

Owing to its powerful performance, deep learning has been widely used in various fields and has gradually become a mainstream model. The visual transformer model has also recently made great breakthroughs in various visual tasks. In order to obtain better clustering performance, we designed a novel method named deep transformer-based cluster (DTCluster) based on visual transformer and K-Means. The specific steps are as follows.

The visual transformer feature extractor is used to extract the features of an image and convert the image into a feature vector. To perform K-Means clustering based on the above characteristics, first set the number of clusters *K*, randomly select *K* data points from the dataset as the centroid according to the set *K* value, and calculate the difference between it and each centroid for each point in the dataset. Then, the point in the dataset is divided into the set of the centroid with the closest distance. After all the data points are put into the set, there are *K* sets in total. Then, the centroid of each set is recalculated. The distance between the new centroid and the original centroid is calculated. If it is less than a set threshold, it means that the position of the centroid has changed slightly and is stable. The algorithm is terminated and the final result obtained. If the distance between the new centroid and the original centroid exceeds the set threshold, the centroid will be recalculated and iterated until the centroid stabilizes. Finally, a cluster label is assigned to each logo image. According to the clustering result, that is, the cluster label of each image, use it as the supervision information to retrain the feature extractor. The above process is iterated until the preset number of iterations is reached and the final clustering result obtained. The contributions of this article are as follows:

- We built a dataset of logo images with various styles, facilitating the research on logo images.
- We proposed a machine learning method for automatic classification of various style images and designed a deep transformer-based cluster (DTCluster) based on visual transformer and K-Means. Based on the basic theory of logo design, we analyzed the aesthetic characteristics of the images in different clusters.
- We experimentally compared standard clustering methods and verified the effectiveness of our proposed method.

The rest of this paper is organized as follows: Section 2 is a review of related work.  Section 3 describes our clustering algorithm and training method.  Section 4 discusses the experimental results and aesthetic analysis of logo image vision.  Section 5 concludes this study.

## 2. Related Work

In this section, we review the related work from the three aspects of computing aesthetics, image feature extraction, and clustering algorithm.

### 2.1. Computational Aesthetics

Taking the logo data as the research object, White et al. [15] divided the spatial arrangement of graphic elements of icons into balance, harmony, change, rhythm, movement, and proportion according to the design criteria and calculated the aesthetic feel of the logo. Henderson et al. [16] learned professional designers' understanding of aesthetics and prepared for aesthetic perception in advance. Galanter et al. [17] and Zhang et al. [18] applied design criteria for aesthetic evaluation. Wong et al. [19] and Li et al. [20] applied design principles to photographs and paintings. Liao et al. [21] studied the main visual elements in icon design through visual features, including complexity, balance, and repeatability, and calculated the distribution of characteristic variable values for statistical analysis. Wang et al. [22] looked at the color rules in corporate icons by calculating the number of main hues and the correlation between hues, saturation, and lightness. Hang et al. [23] generated diversified logos automatically by extracting shapes and colors in the visual structure of logo images. Saleh et al. [24] found that combining the features of color histogram and gradient histogram is the most effective way to describe stylistic features of infographics. Lauresn et al. [25] proposed a method to automatically understand and recognize the best icon set in the icon space by taking the icon candidate set of different functions as input. Henderson et al. pointed out a number of guiding and reference features for logo selection and design. Reber et al. [26] proposed that the factors affecting the aesthetics of visual objects include symmetry, contrast, complexity, and fluency of perception. These works influenced us to achieve better results, more conveniently and effectively.

In order to obtain different design styles of logo images more conveniently, we envisaged automatic classification of logo images according to similar styles through a machine-learning-based method to help designers easily find logo images with similar styles.

### 2.2. Image Feature Extraction and Clustering

Before using the clustering algorithm to classify the images, we needed to extract the characteristics of the images. As deep learning and convolutional neural networks have achieved widespread success in various visual tasks, image feature extraction methods based on deep learning have become the mainstream. These methods use neural network models to mine deeper and more abstract features of images. No manual participation is required, and it is less affected by light posture.

Image feature extraction includes traditional manual feature method and depth feature method. Lowe et al. proposed a scale-invariant feature transform (SIFT) [27] local feature description, by finding extreme points in the spatial scale of the image and extracting its position, scale, and rotation invariants. Dalal et al. proposed a histogram of oriented gradient (HOG) [28] feature descriptor, which composes features by calculating and counting the histogram of the gradient direction of the local area. Bay et al. proposed a speeded up robust feature (SURF) [29], which uses the determinant value of the Hessian matrix for feature point detection and uses the integral graph to accelerate the calculation, which improves the speed of the previous feature descriptor. For the deep feature method, Simonyan et al. [30] studied the influence of convolutional network depth on accuracy in a large-scale image recognition setting. He et al. proposed a deep residual network (ResNet) [31] that uses residual learning to solve the problem of network degradation and make the network deeper. The network proposed by Saining et al. [32] is an upgraded version of ResNet. In this paper, we considered cardinality as another dimension to improve network performance. We considered it more effective in improving the network. Huang et al. [33] proposed DenseNet, a new network framework that has enriched the CNN system since LeNet. The combination of the convolutional neural network model and the attention mechanism dominated the architecture in the past. Later, Wang et al. [34, 35] tried to replace the convolutional architecture with self-attention and made great progress. However, the ResNet neural network was still the state-of-the-art network architecture at the time. Mahajan et al. [36] presented a study of transfer learning with large convolutional networks trained to predict hashtags on billions of social media images. Once again, the practice has reproved that weakly supervised learning is effective work. Xie et al. [37] also proposed a self-supervised approach (unlabeled data) using convolutional networks, emphasizing adding noise to the training process of the student model. Kolesnikov et al. [38] proposed Big Transfer (BiT), a method to augment pretraining when training deep neural networks.

Visual transformer (ViT) [39] was introduced into the computer vision field from the original transformer [39] in the field of natural language processing. Dosovitskiy et al. [39] proposed visual transformer (ViT), which uses a transformer to build deep models without relying on convolutional networks, thus gaining new progress, and achieved state-of-the-art performance on multiple dataset benchmarks. Liu et al. [40] generalized transformer networks for image processing, which are mainly divided into six categories. Therefore, the transformer method brings many new advances in image processing.

For unlabeled images, deep clustering combines feature learning and clustering to form a unified framework, which directly performs clustering on the original images and achieves good performance. We will introduce the methods of deep clustering based on K-Means to improve the clustering model.

MacQueen et al. [41] proposed a K-Means algorithm. By calculating the distance between each object and each seed cluster center, each object is assigned to the cluster center closest to it. Each time a sample is allocated, the cluster center will be recalculated based on the existing objects in the cluster until a certain termination condition is met. Ester et al. [42] proposed a density-based spatial clustering of applications with noise (DBSCAN) method, which defines clusters as the largest collection of connected points with sufficient density. Their method divides regions with sufficiently high density into clusters, which are arbitrary shape clusters in the spatial database. Rui et al. [43] investigated clustering algorithms for datasets emerging in statistics, computer science, and machine learning.

Yang et al. [44] proposed a joint DR and K-Means clustering approach, recovering a “cluster friendly” latent representation for better clustering data. Alqahtani et al. [45] proposed to embed Deep Convolutional Autoencoder (DCAE) approach to learn feature representations and cluster assignments through DCAEs. Their method consists of clustering and reconstruction objective functions. Time-series clustering is an unsupervised technique for data analysis. Ma et al. [46] proposed Clustering Representation Learning on Incomplete time-series data (CRLI), so that the learned representation has good clustering properties.

Tian et al. proposed DeepCluster [47] which combines end-to-end learning with clustering; at the same time, it learns the parameters of the network and clusters the features of the network output.

Caron et al. [48] presented DeepCluster, a clustering method that jointly learns the parameters of a neural network and the cluster assignments of the resulting features. DeepCluster iteratively groups the features using the standard clustering algorithm K-Means and uses subsequent assignments as supervision to update the network weights. The method yields model benchmarks that are state-of-the-art on unsupervised training of convolutional neural networks on datasets such as ImageNet and YFCC100M.

Zhang et al. [49] proposed an alternate optimization algorithm to solve discrete optimization problems. An adaptive discrete approximate linear method (ADPLM) is proposed to solve the key subproblem of binary clustering center learning. Peng et al. [50] proposed a deep-learning-based subspace clustering method, which simultaneously learns a compact representation using a neural network and a clustering assignment by minimizing the discrepancy between pairwise sample-centers distributions. This is the first work to reformulate clustering as a verification problem. Peng et al. [51] discovered a new prior and proposed a new clustering method based on the new discovery.

Logo databases are commonly used for logo recognition and logo detection. After the rise of deep learning technology, many scholars applied deep convolutional neural network (CNN) to logo recognition [52, 53]. Compared with handcrafted features, deep features can directly learn image features from data [54].

### 2.3. Logo Image Database

Logo image database, in the field of computer vision, is often used for logo detection and recognition. Commonly used publicly available logo databases are as follows: BelgaLogos database [55], the first benchmark for logo detection, is used to detect logos; FlickrLogos-32 [2], a larger logo database for target detection, proposes a highly effective and scalable framework for recognizing logos in images; LOGO-Net [56] is used for brand recognition and logo detection, exploring several emerging deep region-based convolutional network techniques; WebLogo-2M [57] is a large-scale logo database and proposes a novel incremental learning approach, SLST; PL2K [58] proposes a class-agnostic logo detector as a general detector; Logo-2K+ [59], a database for logo image recognition, contains large-scale complex real scene data, more than FlickrLogos-32 and LOGO-Net in the category of logos; LogoDet-3K [9] is used for logo detection and recognition and is the largest logo detection database with complete annotations.

## 3. Methods

We built our own logo dataset and used a clustering algorithm to automatically divide logo images to explore their similarities in design.

### 3.1. Introducing Self-Built Dataset

We create a new logo dataset, JN Logo, for the style grouping tasks, which can also be used for logo recognition and similarity retrieval. It includes 14922 logo images. Logo images are crawled from world-renowned logo websites, containing more than 50 categories of content, including more than 90 different countries and regions, most of which are corporate brand logos. To obtain better performance, our database has no landmark images of real scenes, and this paper only uses the self-built database to solve the style grouping problem.

Given a logo database, we need to use a clustering algorithm to divide the logo samples in the database into several disjoint subsets, namely, clusters. Each cluster corresponds to some potential concepts. After obtaining the clusters, we conducted an artistic analysis based on the clustering results and explored the internal design ideas.

### 3.2. Deep Transformer-Based Cluster

This method is mainly composed of feature extraction by feature extractor, K-Means clustering, and pseudo-label retraining feature extractor. The three processes are iterated until the preset number of iterations is met. We will describe the above process in detail later.

#### 3.2.1. Feature Extraction Process

**Figure 1:**
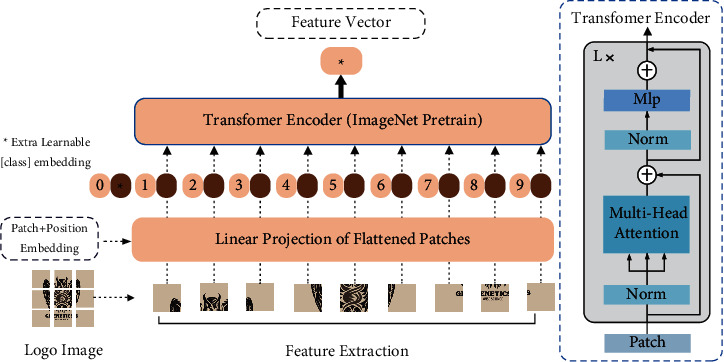
Schematic diagram of feature extraction: first, the original logo image is divided into *N* images of the same size, with each size embedded through linear projection to obtain the embedding vector.

As shown in Figure 1, we first extracted all logo images using a large-scale pretrained visual transformer (ViT) model: the images were standardized, quantized, and scaled up. The standardization was done using a *Z*-Score and the mean and variance of ImageNet datasets.

(1) $$\begin{array}{c} z=\frac{x-\mu }{\sigma }, \end{array}$$

where *x* represents an image and *μ* and *σ* represent the mean and variance, respectively.

As shown in Figure 1, the image with size *H∗W∗C* (height, width, and number of channels) is divided into *NP∗P* image blocks. *N* represents the number of image blocks and *P* represents the size of the image blocks; that is, the size becomes *N∗*(*P*2)*∗C*, where *N*=(*H∗W*)/*P*2. Finally, *N* image blocks are input into the transformer model. We defined the input as *Z*_0_, which consisted of category embedding *X*_class_, embedding vector obtained by linear projection of *N* image blocks, and position embedding *E*_pos_.

Position embedding is a learnable one-dimensional vector used to represent the position of image blocks. Category embedding is the representation of image category.

(2) $$\begin{array}{c} z_{0}=\left[x_{\text{class}};x_{p}^{1}E;x_{p}^{2}E;\ldots ;x_{p}^{N}E\right]+E_{\text{pos}},\;E\in {\mathbb{R}}^{\left(P^{2}.C\right)\times D},\;E_{\text{pos}}\in {\mathbb{R}}^{\left(N+1\right)\times D},\\ z_{\ell }^{\prime }=\text{MSA}\left(\text{LN}\left(z_{\ell -1}\right)\right)+z_{\ell -1},\;\ell =1,\ldots ,L,\\ z_{\ell }=\text{MLP}\left(\text{LN}\left(z{}^{\prime }\ell \right)\right)+z{}^{\prime }\ell ,\;\ell =1,\ldots ,L,\\ y=\text{LN}\left(z_{L}^{0}\right). \end{array}$$

A representation *y* of the final image was obtained through *L*, a transformer encoder, where MSA is the multiplex attention mechanism, LN the layer norm, and MLP the multilayer sensor. *z*_*ℓ*_ is the output of layer *ℓ* transformer. Finally, images are transferred to feature vectors of 1024 dimensions through this model.

At the same time, the embedding vector of the image position was added to a learnable category embedding vector. A vector representation of the original image is then obtained through the transformer model.

#### 3.2.2. Clustering Process

We used a K-Means algorithm for image clustering, and the steps were as follows:

(1) The features extracted in Section 3.2.1 were taken as clustering features, and an appropriate *K* value was selected, where *K* was the final clustering of all images.
(2) According to the set *K* value, *K* data points were randomly selected from the dataset as the center of mass, which represented the center point of the cluster. The random algorithm for selecting the center of mass was the K-Means++ algorithm. We assumed that *n* initial cluster centers had been selected, 0 < *n* < *K*0 < *n* < *K*; then, when selecting the *n*+1 cluster center, the farther the point from the current *N* cluster center, the higher the probability that it was selected as the + cluster center.
The distance we used here was the Euclidean distance as the distance measure, and the distance between samples *A* and *B* is expressed as follows:
  (3) $$\begin{array}{c} \mathrm{dist}\left(A,B\right)=\sqrt{\overset{m}{\underset{i=1}{\sum }}{\left(A_{i}-B_{i}\right)}^{2}}, \end{array}$$
where *m* is the dimension of the sample and *A*_*i*_ and *B*_*i*_ are the *i*-th components of samples *A* and *B*, respectively.
(3) For each point in the dataset, we calculated the distance from each centroid and divided the point in the dataset into the set to which the nearest centroid belong. After grouping all data points into the set, there were a total of *K* sets, and then we recalculated the centroid of each set. The distance between the new and original centroids was calculated. If it was less than the set threshold, then the centroid position did not change much and was stable. The algorithm was terminated and the final result obtained. If the distance between the new and the original centroid changed beyond the set threshold, repeat steps 2-3 until the centroid is stable.

#### 3.2.3. Pseudo-Label Retraining Process

Through the clustering process in Section 3.2.2, we obtained the cluster labels of all logos. These pseudo-labels were used as supervisory information for supervised training of feature extraction, namely, the visual transformer model. In other words, the cluster labels of logos were used as its classification labels to further optimize model parameters through classification training of the model. Specifically, the output of the feature extractor was mapped to the category space through a classification layer, named the full connection layer, and activated by the softmax activation function to obtain the probability of *K* categories. The model parameters are optimized through the backpropagation of the cross entropy loss function, which can be expressed as follows:

(4) $$\begin{array}{c} L_{c}=-\sum_{i=1}^{K}y_{i}\log\left(p_{i}\right), \end{array}$$

where *p*_*i*_ represents the probability that a logo belongs to category *i*. *y*_*i*_ is an indicator variable. When category *i* is the correct category of the sample, *y*_*i*_ is 1; otherwise, *y*_*i*_ is 0.

**Figure 2:**
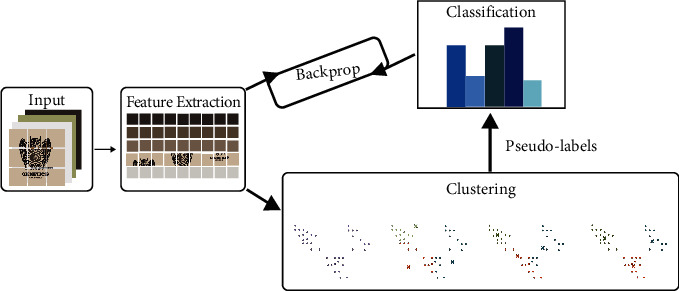
Schematic diagram of DTC method. Images are transformed into features by a feature extractor and then clustered, and the feature extractor is retrained with the clustering results. Finally, the process is constantly iterated to obtain reliable clustering results.

As shown in Figure 2, after the training, we got the new feature extractor parameter, which we fine-tuned using a cluster label, for better clustering performance. After obtaining the new feature extractor model parameters through several iterations, we used the new feature extractor to extract features from logo images; that is, we repeated the feature extraction process. We also repeated the clustering process to obtain new cluster tags, continued to train better feature extractors until the number of iterations reached the preset value, and stopped iteration. We used the last clustering result as the final clustering result.

## 4. Experiment and Analysis

In this section, first, we compare the proposed method with other clustering modalities on logo dataset and analyze the influence of some key parameters. Then, we conduct an aesthetic analysis of different styles of visual features on the experimental results. The feature extraction process was completed on Nvidia Tesla P100 GPU, and clustering process was completed on Intel Core I9-10900K CPU.

### 4.1. Experiment Setting

In the process of image feature extraction, we resized the image to 224 ^∗^ 224 and set the number of small image blocks segmented to 16 ^∗^ 16. We used two internal indicators of clustering performance, DB Index (Davies–Bouldin Index, abbreviated as DBI) and Dunn Index (abbreviated as DI), where DBI can be expressed as follows:

(5) $$\begin{array}{c} \text{DBI}=\frac{1}{k}\sum_{i=1}^{k}\underset{j\neq i}{\max}\left(\frac{\text{avg}\left(C_{i}\right)+\text{avg}\left(C_{j}\right)}{d_{\text{cen}}\left(\mu_{i},\mu_{j}\right)}\right),\\ \text{s.t. avg}\left(C\right)=\frac{2}{\left|C\right|\left(\left|C\right|-1\right)}\sum_{1\leq i\leq j\leq \left|C\right|}\text{dist}\left(x_{i},x_{j}\right),\\ d_{\text{cen}}\left(C_{i},C_{j}\right)=\text{dist}\left(\mu_{i},\mu_{j}\right), \end{array}$$

where *μ* represents the center point of cluster *CC*; *μ*=1/|*C*|∑1 ≤ *i* ≤ |*C*|*X*_*i*_,  avg(*C*) represents the average distance between samples in cluster *CC*; and *d*_cen_(*C*_*i*_, *C*_*j*_) represents the distance between two center points.

DI is expressed as follows:

(6) $$\begin{array}{c} \text{DI}=\underset{1\leq i\leq k}{\min}\left\{\underset{j\neq i}{\min}\left(\frac{d_{\min}\left(C_{i}C_{j}\right)}{\underset{1\leq l\leq k}{\min}\text{diam}\left(C_{1}\right)}\right)\right\},\\ \text{s.t. diam}\left(C\right)=\underset{1\leq i\leq j\leq \left|C\right|}{\max}\text{dist}\left(x_{i},x_{j}\right),\\ d_{\min}\left(C_{i},C_{j}\right)=\underset{x_{i}\in C_{i},\;x_{j}\in C_{j}}{\min}\text{dist}\left(x_{i},x_{j}\right), \end{array}$$

where diam(*C*) is the maximum distance between samples in cluster *C*, and *d*_min_(*C*_*i*_, *C*_*j*_) represents the distance between the nearest samples in clusters *C*_*i*_ and *C*_*j*_.

### 4.2. Comparative Experiment

On our logo dataset, we compared different clustering methods based on K-Means, including traditional SIFT feature extraction method, convolutional neural network feature extraction method, and visual transformer feature extraction method. We have also carried out the experiment of adding iterative process training to CNN feature extraction method. The results are shown in Table 1; the smaller the DBI value, the better, and the larger the DI value, the better; the proposed DTCluster method is optimal in both DBI and DI, which can get the best result.

### 4.3. Hyperparameter Analysis

We analyze two key parameters in the proposed method, namely, the number of clusters and block size in the feature extractor.

As shown in the experimental results in Table 2, we used different block sizes for training, including 8 *∗* 8, 16 *∗* 16, and 32 *∗* 32, but for the other sizes, the models could not be used due to the lack of pretrained models on ImageNet. It can be seen that the best results can be achieved when the block size is 8 *∗* 8, but because the sequence length of transformer is inversely proportional to the square of block size, when the block size is 8, it will bring more computation.

The selection of K hyperparameters in K-Means was determined mainly based on some objective indicators. We used multiple *K* values for clustering and determined that *K* = 6 is a better number of clusters by comparing the DBI values. As shown in Table 3, although the DBI when *k* = 2 and 3 is better than that when *k* = 6, we want to explore more design styles. Relatively, *k* = 6 not only ensures a certain number of clusters, but also achieves a better DBI.

### 4.4. Aesthetic Analysis of Results

We obtained the clustering center of each cluster by calculating the average value of each cluster sample and analyzed the images near the cluster center. In terms of the logo color, graphics, structure, and style of the four aesthetic characteristics of the analysis, the following is the style of cluster centers 0–5:

  Center 0 is a logo design with abstract geometric shapes and English words, mainly blue and green.
  Center 1 is the red abstract graphics with Chinese characters, English words collocation logo design.
  Center 2 is a logo design with background images, illustrative images, and English words.
  Center 3 is a logo design with some red abstract graphics and English words.
  Center 4 is a logo design with background, high saturation abstract graphics, and English words.
  Center 5 is the logo design of some illustrative graphics and English words.

**Figure 3:**
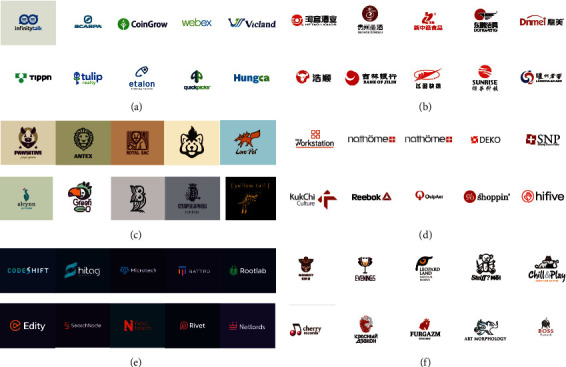
We listed 10 images near the center of each cluster and also conducted aesthetic analysis of four visual features: (a) center 0, (b) center 1, (c) center 2, (d) center 3, (e) center 4, and (f) center 5.

Figure 3 shows the 10 images in the cluster center. We made an aesthetic analysis of the logo images based on the four visual features of centers 0–5: color, graphics, structure, and style.

Center 0. (1) Color: The colors are cool, mainly blue and green, with two adjacent colors. Blue represents calm and rational. Light green, medium green, and dark green represent hope and vitality. Color saturation is moderate, with slightly lower brightness. The visual sense is mature, contracted, and composed. The lower lightness and brightness give a low-key feeling of introversion. (2) Graphics: Being abstract geometric circle, the logo of the graph is curve based. The circular geometry has a lightness and ease of feeling. (3) Structure: Round logo and round English words go together harmoniously. Rectangular and triangular logos and square fonts work together harmoniously. (4) Style: The shape is unique, and the graph is concise and rich in variation, with fashion and simple style characteristics and with a certain rational feeling.

Center 1. (1) Color: Bright red and deep red based warm colors represent enthusiasm, positive, fighting, and optimistic meaning, having a strong appeal, with high saturation, rich color, moderate brightness. The combination of bright red and black symbolizes enthusiasm and positivity. The combination of deep red and black symbolizes maturity and vitality. The combination of dark red and dark blue is easy to identify. (2) Graphics: They are mainly abstract geometric figures, with circular, curvilinear modeling. The circular shape gives people a smart, full, and mellow feeling in the vision. Chinese characters symbolize traditional Chinese culture and are easy to recognize. (3) Structure: The circular image is matched with Chinese characters and English words. The overall composition is left and right structure and upper and lower structure, with a sense of order and rhythm. (4) Style: It is enthusiastic and positive visual perception of traditional culture. Bright red is the main representative color of Chinese traditional culture, with a sense of justice and sincerity and the sense of the times. It is suitable for traditional Chinese industries in application scenarios.

Center 2. (1) Color: There is background color, with no pattern, moderate saturation, and moderate brightness. Color combination is more harmonious and comfortable. It gives a person sedate, serene feeling. (2) Graphics: Illustrative form of modeling is used; visual expression is clear and easy to identify. (3) Structure: Structurally, the graphics and English words match harmoniously, mainly the upper and lower structure. The overall structure maintains a pictorial feel, which is very decorative. (4) Style: The logo has artistic style and strong decorative aesthetic feeling.

Center 3. (1) Color: These two colors are bright, with high saturation and high lightness, symbolizing a positive, optimistic and enthusiastic meaning, with a strong appeal. The font is black or gray, and black has a cool, technological feel to it. A combination of black and red was visually stimulating. Red and gray combination, with the ornament of gray, more set off the bright red. (2) Graphics: They are abstract geometric figures, including circles, squares, and triangles. Squares and triangles are visually stable, with a sense of security and order. However, circles have flexibility, unlike squares and triangles. The font design added interesting symbols, not rigid. (3) Structure: They are structurally neat and belong to the typical up and down, left and right structure. (4) Style: They are characterized by a warm, positive style. The combination of red and black or gray is suitable for business enterprise logo. These visual feelings show that they are suitable for the Internet industry and science and technology industry.

Center 4: (1) Color: They all have dark backgrounds, mainly dark purple, dark blue, dark green, dark green cool tones. The colors of the graphics are light colors with higher lightness. Light green and light blue belong to cool colors; orange red and rose red belong to warm colors. Cool color background and cool color graphics combined are more harmonious. There is strong contrast with the graphic combination of warm colors. Integral color has sedate feeling. (2) Graphics: Abstract graphic symbols blend with the English alphabet. English font is used according to the graphic symbols to do the corresponding edge adjustment, and the overall look is very harmonious. (3) Structure: The proportion gap between graphics and English fonts is very small and integrated, and the overall structure is very coordinated. There is a sense of visual thickness, and the overall structure is relatively stable. (4) Style: They have a sensible, sedate visual style.

Center 5. (1) Color: The colors are mainly dark red, bright red, black, brown, and khaki. Each figure is composed mainly of monochrome. The saturation is moderate, and the brightness is low, so the color is more gentle. There is a certain gray level in the color, and the overall color has a lightsome aesthetic feel. (2) Graphics: Their graphics are also in the style of illustration, but the design is simpler. The overall feeling is relaxed and full. They have a very cartoon-like shape, full of children's fun. (3) Structure: The simple structure belongs to the top-down infrastructure. (4) Style: They have an artistic style of illustration. It has a unique artistic atmosphere and rich sense of interest and lightness. Their graphics pay attention to details and contain philosophy. Their shapes are more concise; they bring light, interesting visual experience.

### 4.5. Comparative Analysis of Experiments

In order to further verify the validity of our clustering algorithm. We experimented with our data using three other clustering algorithms: the density-based spatial clustering of applications with noise (DBSCAN), spectral clustering, and hierarchical clustering. Experiments showed DBSCAN as the most common and effective method among the three methods. The results of DBSCAN algorithm were used for comparative analysis.

DBSCAN is a representative density-based clustering algorithm, which defines the cluster as the largest set of points connected by density, can divide the region with high enough density into clusters, and can find clusters of arbitrary shape in the spatial database of noise. In the experiment, we set the distance threshold eps to 0.5 and set the threshold of sample number to 5.

Spectral clustering is a kind of method based on the graph theory. It could recognize any shape of the sample space and restrain it to the most global solution. Its basic idea is to use the similarity array of the sample data to extract the feature variables and then cluster them. In the experiment, we set the number of clusters to 6.

Hierarchical clustering creates a hierarchical nested clustering tree by calculating the similarity between data points of different categories. In the clustering tree, the original data points of different categories are the lowest layer of the tree, and the top layer of the tree is a root node of clustering, which includes two clustering methods: bottom-up and top-down. In this experiment, we use the bottom-up method.

We analyzed the images near the cluster center. The following is the style of cluster centers 0–8:

   Center 0 is artistic and relaxing, but the fonts were very messy, with some in Chinese and others in English.

  Center 1 is simple, heavy, and monochromatic and in English, but the color distribution is messy.
  Center 2 has a dark background with a sense of space and weight in English fonts. The color of the logo image is relatively simple.
  Center 3 is a layered Chinese font.
  Center 4 is a combination of graphics and English fonts. It occasionally has background colors and is not uniform.
  Center 5 has a dark background, with artistic and heavy visual feel.
  Center 6 is an artistic, relaxing visual experience. The colors are warm. The font of the logo is curvy and flexible with the graphics. But it has logo images on a light background that are not uniform.
  Center 7 is artistic and light visual experience. Center 7 and center 8 have great similarities.
  Center 8 is something artistic and light. The colors are cool. They have obvious features of larger graphics.

**Figure 4:**
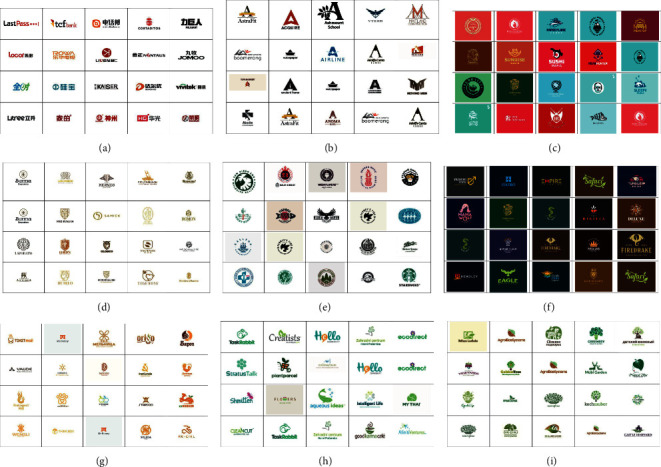
Since DBSCAN is assumed to have a noisy space, nine different logo design styles were generated by clustering after removing the noisy images. We show the images near the cluster center: (a) center 0, (b) center 1, (c) center 2, (d) center 3, (e) center 4, (f) center 5, (g) center 6, (h) center 7, and (i) center 8.

As seen from Figure 4, there are obvious stylistic characteristics between styles. However, the image styles for center 3 and center 6 are very similar, as are center 7 and center 8 images. There are repeated styles in these images. Compared to other methods, the style of our method is not repeated.

Experimental results show that our method effectively distinguishes different styles of logos, but there are still some problems. For example, although we collected X logos, we did not comprehensively cover all styles.

## 5. Conclusions

In this study, we built a logo dataset with various styles and designed a deep clustering algorithm based on visual transformer and K-Means. We clustered logo images to further explore design styles of the clustering results, and analyzed with aesthetic empirical knowledge to evaluate from four aesthetic characteristics. This study was an attempt to automatically extract the internal visual design style of logo images, which changed the previous artificial preset style and designed an automatic machine learning framework. Its practical significance is that the analysis of logo styles assists designers in the conception and design of logo images.

However, the proposed method still has some limitations. First, the logo images in the dataset cannot cover all design styles. We need to continue to increase the size of the dataset. At the same time, our model lacks some external index evaluation. The clustering algorithm currently used is based on similarity or density but does not add some additional prior knowledge, such as different logo colors and shapes. In the future, we will continue to enrich the content of our dataset, and introduce external indicators through some annotation means to make a more comprehensive evaluation of our clustering method.

## Figures and Tables

**Table 1:** Performance comparison of internal indicators in different clustering methods.

Method	DBI ↓	DI ↑
SIFT + K-Means	4.009	0.093
CNN + K-Means	3.642	0.132
ViT + K-Means	3.565	0.167
DTCluster (CNN)	1.567	0.171
**DTCluster (ViT, ours)**	0.904	0.189

**Table 2:** Comparison of the DBI and DI of block size.

Method	DBI↓	DI↑
ViT (32 ^*∗*^ 32) + K-Means	3.587	0.164
ViT (16 ^*∗*^ 16) + K-Means	3.565	0.167
ViT (8 ^*∗*^ 8) + K-Means	2.916	0.175
DTCluster (32 ^*∗*^ 32)	0.947	0.188
DTCluster (16 ^*∗*^ 16)	0.904	0.189
DTCluster (8 ^*∗*^ 8)	0.751	0.194

**Table 3:** Clustering using various *K* and DBI values.

*K*	2	3	4	5	6	7	8	9	10
DBI	2.030	2.837	3.432	3.585	3.565	3.674	3.589	3.599	3.644

## Data Availability

The data used to support the findings of this study are currently under embargo while the research findings are commercialized. Requests for data, 6/12 months after publication of this article, will be considered by the corresponding author.
